# Fabrication-Method-Dependent Excited State Dynamics in CH_3_NH_3_PbI_3_ Perovskite Films

**DOI:** 10.1038/s41598-017-16654-1

**Published:** 2017-11-28

**Authors:** In-Sik Kim, Cheol Jo, Rira Kang, Dong-Yu Kim, Seong-Jin Son, In-Wook Hwang, Do-Kyeong Ko

**Affiliations:** 1 Department of Physics and Photon Science, Gwangju Institute of Science and Technology, Gwangju, 61005 Republic of Korea; 2 Radiation Research Division for Industry & Environment, Korea Atomic Energy Research Institute, Jeongeup, Jeollabuk-do 56212 Republic of Korea; 3 School of Materials Science and Engineering, Gwangju Institute of Science and Technology, Gwangju, 61005 Republic of Korea; 4 Advanced Photonics Research Institute, Gwangju Institute of Science and Technology, Gwangju, 61005 Republic of Korea

## Abstract

Understanding the excited-state dynamics in perovskite photovoltaics is necessary for progress in these materials, but changes in dynamics depending on the fabrication processes used for perovskite photoactive layers remain poorly characterised. Here we report a comparative study on femtosecond transient absorption (TA) in CH_3_NH_3_PbI_3_ perovskite films fabricated by various solution-processing methods. The grain sizes and the number of voids between grains on each film varied according to the film synthesis method. At the low excitation fluence of 0.37 μJ cm^−2^, fast signal drops in TA dyanmics within 1.5 ps were observed in all perovskite films, but the signal drop magnitudes differed becuase of the variations in charge migration to trap states and band gap renormalisation. For high excitation fluences, the buil-up time of the TA signal was increased by the activated hot-phonon bottleneck, while the signal decay rate was accelerated by fluence-dependent high-order charge recombination. These fluence-dependent dynamics changed for different perovskite fabrication methords, indicating that the dynamics were affected by morphological features such as grain sizes and defects.

## Introduction

Organic-inorganic lead halide perovskite semiconductors are among the most promising alternatives for third-generation photovoltaics because they show exceptional advantages of low-cost straightforward fabrication, high absorption in the visible range, small exciton binding energy, and flexibility^1–3^. With the recent extensive study of these systems, the power conversion efficiency (PCE) of perovskite solar cells (PeSCs) has explosively increased dramatically from 3.8% in 2009 to >20% since 2015^4–6^. The high PCEs of PeSCs are attributed to their outstanding photo-physical properties, including the long lifetimes and diffusion lengths of photoexcited carriers and excellent carrier transport performance. These are characterised by the time scales of charge generation, recombination, and transfer dynamics assessed using ultrafast spectroscopic measurements^7–9^.

One effective methodological approach to enhance the performance of solution-processed PeSCs is the fabrication optimisation of the perovskite photoactive layer, achieved by controlling the composition and/or condition of the precursor solution, the types of solvents and additives, and the processing environment. These factors for fabrication engineering are known to affect the photovoltaic performances of PeSCs by changing the uniformity of micromorphology, grain size, and crystallinity in the perovskite film^10,11^.

The dependence of PCE on perovskite surface morphology was successfully demonstrated by increasing the sizes of cuboids formed using different concentration of CH_3_NH_3_I (methylammonium (MA) iodide; MAI) precursor solution by Im *et al*.^12^. Grancini *et al*. revealed that the crystalline domain sizes in hybrid lead-halide perovskite films depends on the fabrication procedure; thus, the dynamics of excited species including free carriers and excitons are influenced by the crystal size^13^. Matsumoto *et al*. reported alteration in the photovoltaic parameters with photodegradation-induced surface roughness^14^. Although the correlation between perovskite morphology and photovoltaic parameters were confirmed in these cases, others have indicated that the surface coverage of the perovskite affected the short-circuit current density (*J*_*SC*_) but that the effects of the morphology on the PCE were less clear^15^. In addition, Li demonstrated that the photoluminescence decay lifetime did not change with variations in morphology above a certain grain size^16^.

Our previous study also demonstrated ambiguity in the relationship between morphology and device performance^17^: In the study, we explored the effects of various deposition methods on the performances of solution-processed planar CH_3_NH_3_PbI_3_ (MAPbI_3_) PeSCs using single- and two-step depositions. The grain size distribution was generally similar for each perovskite, but slight differences occurred; the number of grains larger than 300 nm in the film increased in the order of perovskites named CHP (single-step deposition of the precursor solution with N-cyclohexyl-2-pyrrolidone as an additive), CBdrp (single-step deposition of the precursor solution by dropping chlorobenzene, a poor solvent, during the spin-coating procedure), and IFF (two-step deposition including interdiffusion of the MAI precursor on PbI_2_ film). However, despite having the small grains, the CHP-based PeSC showed the best performance, with averaged photovoltaic parameters including a *J*_*SC*_ of 14.31 mA cm^−2^, open-circuit voltage (*V*_*OC*_) of 0.86 V, and fill factor (*FF*) of 73%, leading to a PCE of 9.07%. The solar cell device including CBdrp film had a *J*_*SC*_ of 13.80 mA cm^−2^, *V*_*OC*_ of 0.78 V, and *FF* of 73%, leading to a PCE of 8.03%. Finally, the IFF solar cell had a *J*_*SC*_ of 13.38 mA cm^−2^, *V*_*OC*_ of 0.80 V, and *FF* of 67%, leading to a PCE of 7.19%^17^. The responses of these three perovskite films on silicon substrates to terahertz waves indicated the highest free carrier accumulation at the CHP perovskite/silicon interface^18^, but the photophysical properties of these perovskites were poorly known.

Therefore, in this work, the ultrafast responses of MAPbI_3_ perovskite films fabricated by CHP, CBdrp, and IFF are investigated and compared using femtosecond transient absorption (TA) spectroscopy in order to examine the mechanisms underlying PeSC functionality by relating the excited-state dynamics to the film morphology. The TA spectrometer setup is briefly explained in the Supporting Information and depicted elsewhere^19^.

## Results and Discussion

**Figure 1:**
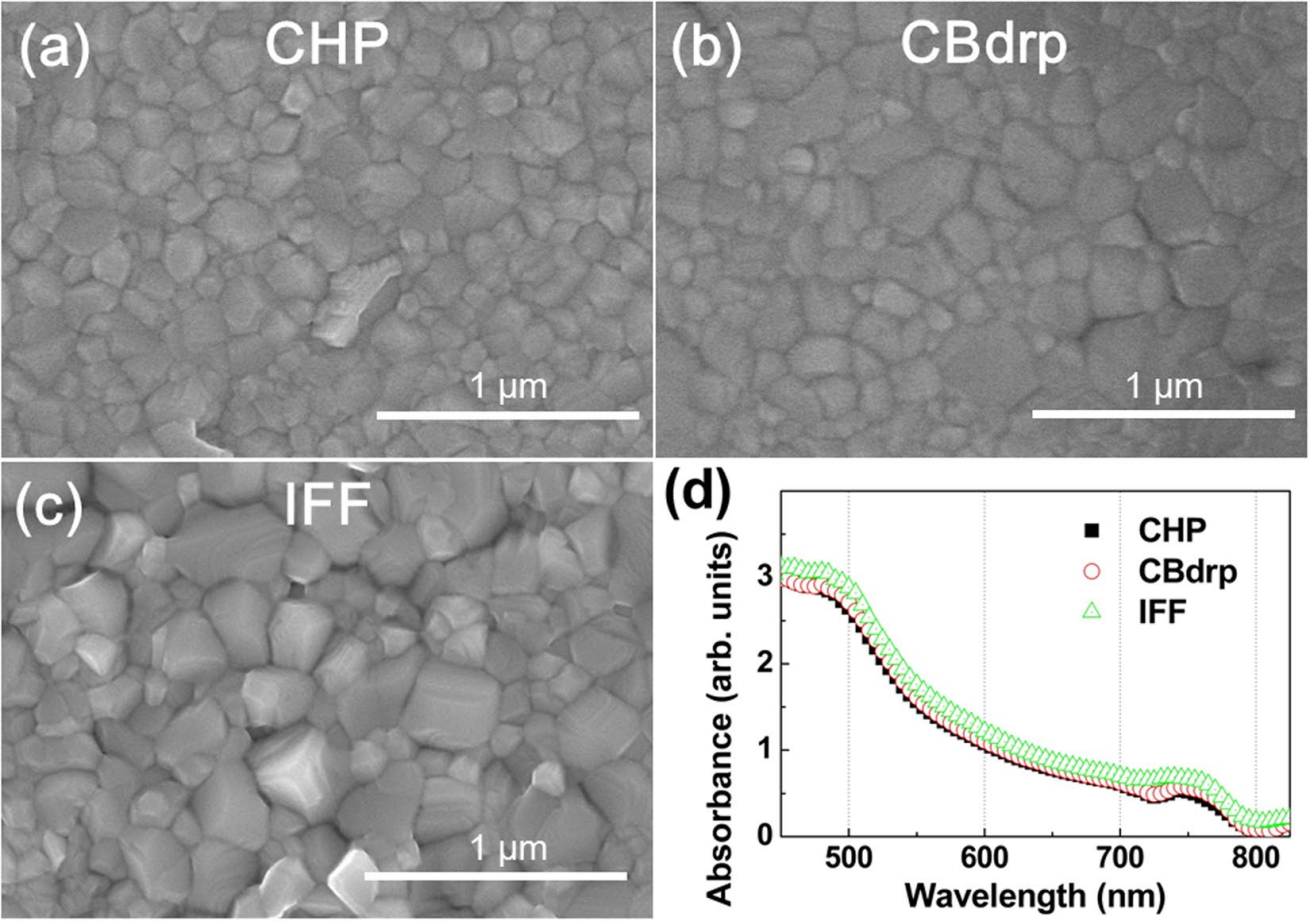
Scanning electron microscopy images of MAPbI_3_ films fabricated on PEDOT:PSS/ITO by (**a**) CHP, (**b**) CBdrp, and (**c**) IFF methods, and (**d**) steady-state absorption spectra of each perovskites coated on glass substrate.

Figure 1(a–c) show scanning electron microscopy (SEM) images of the three perovskite films. As shown in our previous work^17,18^, while the morphology of the CHP film mainly comprises small grains and shows complete surface coverage, larger grains and intergranular voids appear in the CBdrp and IFF films.

Figure 1(d) shows the steady-state absorption spectra of the MAPbI_3_ perovskite films. All films show two conspicuous positive curvatures at comparable wavelengths and absorption-band edges located at ~780 nm. These similarities among the absorption spectra indicate that the energy levels, excitonic and continuum contributions to the absorption, binding energies, and band gaps are not influenced by the sample preparation methods used. The perovskite films are approximately 190 nm, 230 nm, and 330 nm in thickness for CHP, CBdrp, and IFF films, respectively^17^; therefore, the absorbance of the IFF film may be slightly larger than those of the CHP and CBdrp films.

Figure S1(a–c) present the TA spectra of the perovskite films after 400-nm excitation with the fluence of 2 μJ cm^−2^ at several pump-probe delays. For short time delays, each film exhibits a broad positive band from the photo-bleaching (PB) of both the band edge and near-band-edge regions by state-filling with hot carriers^20^. In addition, the films also show negative TA signals at energies below the band gaps, indicating that carriers are excited to transiently unoccupied states formed by band gap renormalisation (BGR)^20^. As the pump-probe delays are increased, the short-wavelength tails of the PB bands are changed from positive to negative values; the PB bands are also slightly red-shifted and the negative TA signals below the band gap disappear. These features reflect the instantaneous evolution of excited energy states during hot-carrier cooling and BGR^20–22^. Because the TA spectral features of the CHP, CBdrp, and IFF films are almost identical, the TA kinetics at the PB peak was examined to obtain more details of the excited-state nature.

**Figure 2:**
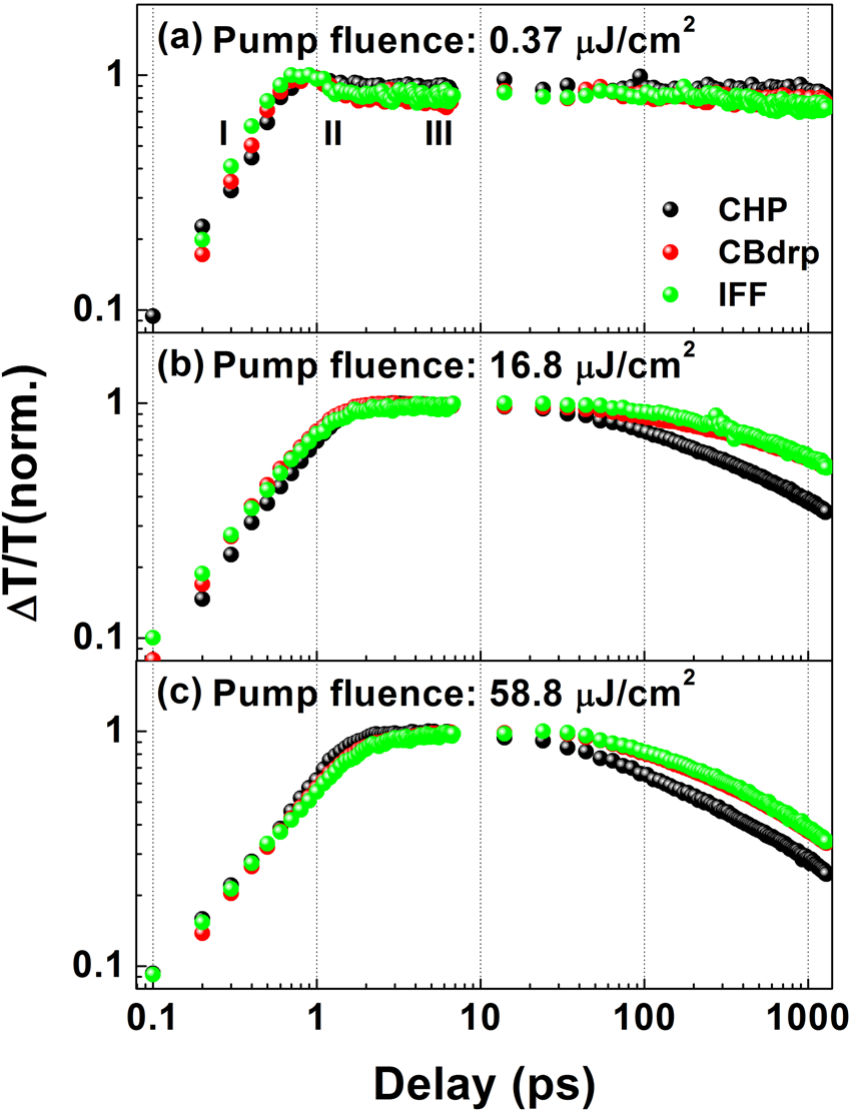
Normalised TA kinetics of MAPbI_3_ films fabricated by CHP, CBdrp, and IFF fabrication methods, probed at 760 nm with 400-nm excitation at the fluences of (**a**) 0.37 μJ cm^−2^, (**b**) 16.8 μJ cm^−2^, and (**c**) 58.8 μJ cm^−2^. Fits for the decays in (**b**) and (**c**) are shown in Fig. S4.

Figure 2 shows the TA decay kinetics of the perovskites formed by the CHP, CBdrp, and IFF methods, probed at 760 nm after 400-nm excitation with several fluences. Within the first 10 ps, the TA decay dynamics at the low excitation fluence of 0.37 μJ cm^−2^ (Fig. 2(a)) indicates three distinct phases: build-up of the TA signal within 1 ps (phase I), a slight drop to a plateau within 0.5 ps (phase II), and the long plateau (phase III). Phases I and II include the initial excited-state dynamics of the formation and cooling of the hot carriers. Just after phase II, the relative intensities of the peak TA values differ for the CHP (0.88 ± 0.025), CBdrp (0.80 ± 0.025), and IFF (0.81 ± 0.029) films, as determined statistically from 20 data points obtained at the end of phase II. These differences are attributed to the presence of trap states^23^, which can induce charge transfer from the band edge of excited states to below-gap trap states. The smallest amount of trap-mediated kinetics was predicted for the CHP film because it show full coverage and closely adjacent grains^24–26^. Because the signal drops in phase II can also arise from red shifts in the PB band peak caused by the BGR accompanying the initial hot-carrier dynamics, accordingly, the dynamics in phase II are the result of the simultaneous contributions of trap states and the BGR.

As the excitation fluence is increased (i.e. carrier density is increased), phases I and II disappear from the kinetics, and the time before the TA signal maximizes is decreased to ~2 ps (see Fig. 2(b)). This impeded rise time, indicating the deceleration of the hot-carrier cooling rate, is mainly ascribed to a hot-phonon bottleneck by hot-phonon reabsorption, which is positively correlated with the excited carrier density^20,21^. Thus, the signal growth dynamics are slowed with further increases in excitation fluence, as shown in both Figs 2(c) and S3. In general, hot-carrier cooling rates can be determined by analysing the hot-carrier temperature^20,21^. Thus, the TA spectra in Fig. S1 were fitted with Maxwell–Boltzmann distribution functions and the time-dependent carrier temperature in each perovskite was determined as shown in Fig. S2. The fit results showed that both the initial carrier temperature and the carrier cooling time were increased with the increase of excitation fluence (from 2 μJ cm^−2^ to 58.8 μJ cm^−2^); however, the subtle differences among the carrier cooling curves of the perovskites were difficult to observe because of large deviations from the fitting model. Therefore, we simply compared the early-time dynamics of TA signals in 10% to 90% of the rise time without the fitting procedure using the Maxwell-Boltzmann distribution.

By comparing the rise times of the kinetics (see Table 1), the carrier cooling rates are clearly observed to vary in each perovskite film. The hot-phonon bottleneck is activated in proportion to the initial carrier density; however,the carrier density of the CHP film is larger than those of the other films even at the same excitation fluence because of the higher absorption coefficient of the CHP film. This implies that other factors affect the carrier cooling in addition to the carrier density. Meanwhile, the hot-carrier cooling at high fluence (i.e. carrier densities >10^18^ cm^−3^) depends on the amount of unreacted PbI_2_ remaining in the perovskite film^21^, and the conversion of PbI_2_ to the perovskite is known to directly affect the formation of large crystals^27^. Therefore, we speculate that the cooling dynamics are influenced by the crystal size; this was confirmed by sample-to-sample variability testing of early time dynamics at high excitation fluence (see Fig. S5(a),(b)). Faster rising aspects in the CHP films, compared to those in the CBdrp and IFF films, were reproduced in these tests. However, the rise times of the IFF films varied from sample to sample. The largest-grained perovskites were fabricated using the IFF method, but the size formation was pooly controlled, generating broad distributions for the grain sizes and photovoltaic parameters^17^. Therefore, the morphological heterogeneity among the IFF films may affect the hot-carrier cooling characteristics; likewise, the shortest carrier cooling times in the CHP films can be attributed to these films having the smallest grains.

**Table 1:** Rise times (τ_rise_) were measured from the time required for each TA signal to grow from 10% to 90%; errors for τ_rise_ were obtained from the deviation in several TA datasets from the same sample.

Perovskite fabricated by	Excitation fluence [μJ cm^−2^]	τ_rise_ [ps]	τ_1_ [ps]	τ_2_ [ps]
CHP	16.8	1.3 ± 0.02	96 ± 3 (43%)	724 ± 31 (57%)
	58.8	1.6 ± 0.04	73 ± 2 (51%)	597 ± 23 (49%)
CBdrp	16.8	1.2 ± 0.04	96 ± 6 (25%)	876 ± 38 (75%)
	58.8	2.5 ± 0.04	85 ± 2 (39%)	648 ± 10 (61%)
IFF	16.8	1.2 ± 0.03	130 ± 22 (21%)	861 ± 71 (79%)
	58.8	2.5 ± 0.03	84 ± 5 (36%)	625 ± 25 (64%)

The decay time constants (τ_1_, τ_2_) were determined by bi-exponential decay fits (ΔT/T(*t*) = *A*_1_exp(−*t*/τ_1_) + *A*_2_exp(−*t*/τ_2_)). The numbers in round brackets indicate relative amplitudes of *A*_1_ and *A*_2_ in percentages. Errors for τ_1_ and τ_2_ indicate the deviation between the TA decays and the fitting function.

The TA kinetics at low pump fluence (Fig. 2(a)) showed no significant decay in a time window range from 10 ps to 1.2 ns. This is because the carrier recombination of a perovskite is dominated by free carriers with the recombination times of several tens of nanoseconds^7,22^, which causes difficulties in distinguishing the kinetics within the time window of 1.2 ns. Figure 2(b) and (c) display the TA kinetics of the three perovskites at higher pump fluences, and Fig. S4 and Table 1 show the bi-exponential fits of the TA kinetics and the corresponding time constants, respectively. In all perovskite films, the TA kinetics at high pump fluences is characterised by accelerated decays compared to the dynamics at the fluence of 0.37 μJ cm^−2^. This excitation fluence-dependent bleaching dynamics has been previously reported in various perovskite absorbers and ascribed to fluence-dependent high-order processes, such as bimolecular electron–hole recombination and trimolecular Auger rebombination^28,29^.

As shown in Table 1, the time scale of accelerated decay by high-order processes in the perovskite films is several tens to hundreds of picoseconds, which is markedly longer than that in polymer films^30–32^. These are associated with the previously reported high-order recombination rate constants for perovskite systems (~1 × 10^−10^ to ~1 × 10^−9^ cm^3^ s^−1^)^29,33,34^ and polymers semiconducting materials (~4 × 10^−9^ to ~3 × 10^−8^ cm^3^ s^−1^)^35,36^. Because the organic polymers are low mobility materials, thus they follow Langevin model in which the high-order recombination rate is proportional to the charge carrier mobility. The reaction radius of Langevin type recombination is limited to ~1 nm because the Langevin model assumes electron–hole separation occurs within Coulomb radius^37–40^. On the other hand, non-Langevin recombination arises in some semiconducting materials in which electron-hole pairs are spatially separated^39,40^. As the perovskite system are characterised by weak correlation between the high-order recombination rates and mobility^28,33,40^, the perovskite can be also affected by the non-Langevin recombination. Perovskite grain boundaries govern carrier trap and recombination sites as carrier confining structures^41,42^; therefore, the spatial separation of electron-hole pairs for the non-Langevin recombination in perovskite may be mainly determined by the grain boundaries. Because the grain sizes determine the amount of boundaries and are formed in a range from several hundred nanometres to several micrometres, the reaction dimensions for high-order processes in perovskite films can be significantly increased, inducing relatively slower high-fluence-induced kinetics compared to polymer films. Therefore, more active high-order processes are anticipated in smaller-grained perovskites film than in larger-grained film. And such phenomenon was confirmed by observing faster high-fluence-induced TA dynamics in the CHP film than in the CBdrp and IFF films (see Fig. 2(b) and (c)). In addition, the sample-to-sample variability testing of decay dynamics at high excitation fluence showed that the decay dynamics changed from sample to sample in the IFF films but not in the CHP and CBdrp films (see Fig. S5(c) and (d)). This is ascribed to both the poorly controlled grain sizes when the IFF fabrication method is used and high sample-to-sample reproducibility in the CHP and CBdrp methods.

The fast high-fluence-induced dynamics in the CHP film may also relate to surface morphology. Several studies have shown that complete surface morphology on perovskite films induces more free carriers and reduces trapped species^24–26^. Therefore, the relatively active recombination at high fluences can be anticipated in the CHP film, arising from its complete coverage without defect may increase the chance of high-order recombination involving more free carriers.

**Figure 3:**
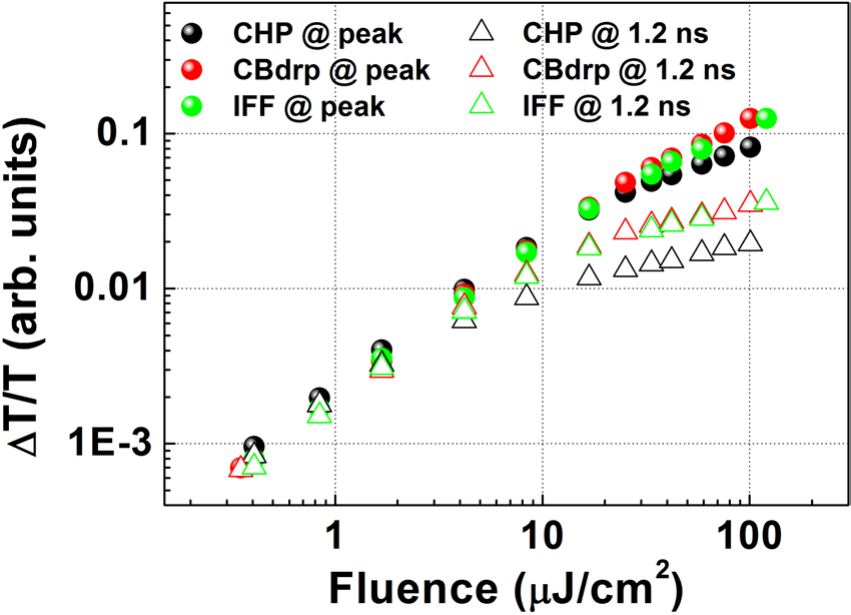
Comparison of excitation fluence dependencies of TA signal intensities probed at 760 nm, extracted from the TA signals in Fig. S6.

As shown in Fig. 2 and Table 1, when the excitation fluence is increased from 16.8 μJ cm^−2^ to 58.8 μJ cm^−2^, the TA kinetics of all films show further acceleration in decay. However, the degrees of decay acceleration in the CBdrp and IFF films are comparatively larger than that in the CHP film. This acceleration trend is also found in the kinetics under the pump fluence of 100 μJ cm^−2^ (Fig. S3). The difference in long-lived TA signals between the CHP and CBdrp (and IFF) films is gradually decreased with increasing fluence. This saturation feature in the CHP film also appears in the excitation fluence-dependent PB intensity. Figure 3 displays the excitation fluence dependence of the 760-nm PB intensity at the peak, as well as at longer time delays (1.2 ns), maintaining a steady variation. The 760-nm TA signal intensity at the peak increases nonlinearly with increased the excitation fluence, and the nonlinearity is more prominent in the variations of the TA signal intensities at the 1.2-ns delay. This nonlinearity was previously ascribed to the coexistence of band filling by both excitons and the free carriers, which can saturate the exciton contribution^21,43^. Only band filling prevails in the PB signals at the fluence scale of several microjoules per square centimetre, thus inducing linear absorption at low fluences. However, the band filling contribution can be saturated by the free carriers at increased excitation fluences^21,29,34^. Therefore, the noticeable saturation of the PB signal with increasing excitation fluence in the CHP film may be ascribed to the efficient generation of the free carriers.

## Conclusion

We conducted TA spectroscopy on MAPbI_3_ perovskite films fabricated by the CHP, CBdrp, and IFF methods and demonstrated that the carrier dynamics of the perovskite films depended on the sample fabrication methods. At the low excitation fluence of 0.37 μJ cm^−2^, fast signal drops in TA dyanmics within 1.5 ps were observed in all perovskite films, but the magnitude of the signal drop was smaller in the CHP film than in the other films. Charge migration from the excited band edge to trap states, as well as the BGR, contributed to this fast dynamics. Compared to the early-time dynamics at low excitation fluences, the rise times of the TA signals in the perovskite films were slowed with increasing excitation fluence because of the hot-phonon bottleneck. The grain size appeared to affect to the hot-phonon bottleneck, as demonstrated by the shorter rise time in the smaller-grained CHP film relative to those in the other larger-grained films. Similarly, the rise times showed significant variation in the IFF films, in which the grain size was poorly controlled among the samples. At high excitation fluence, the TA decay rate was accelerated by fluence-dependent high-order recombination phenomena. The high-fluence-induced decay was the fastest in the CHP film with the smallest average grain size and varied in the IFF films having poorly controlled grain sizes, implying that the high-order recombination was also dominated by grain size. In addition, because complete surface coverage can form more free carriers and reduce trapped species, more active free carrier-induced high-order recombination might occur in the CHP films containing no voids between grain boundaries. Accordingly, our results demonstrated the relationship between morphology and carrier dynamics, offering insight toward achieving higher performances in perovskite photovoltaics.

## Experimental Section

The perovskite films were spin-coated on poly(3,4-ethylenedioxythiophene) polystyrene sulfonate (PEDOT:PSS)/indium tin oxide (ITO) for the SEM imaging and on glass substrates for the femtosecond TA spectroscopy using previously reported methods^17^. The excited-carrier dynamics in the perovskite films was studied using the TA spectrometer based on an ultrafast titanium:sapphire amplifier system (Spitfire Ace PA, Spectra-Physics), which generated 35 fs pulses centred at 800 nm with a 1-kHz repetition rate. The wavelengths of the amplified beams were converted to the 400-nm pump and broad band visible probe beams using second harmonic and supercontinuum generations, respectively. The instrument response function was estimated as ~140 fs by measuring the cross-correlation of the pump and probe. The polarisations of the excitation and probe beams were set to the magic-angle configuration (54.7°). The TA experiments were performed under rough vacuum and at temperature of 293 K.

## Electronic supplementary material

Supplementary Information
