# Supplementary material for: Fabrication-Method-Dependent Excited State Dynamics in CH3NH3PbI3 Perovskite Films
Source: Sci Rep. 2017 Nov 28;7:16516. doi: 10.1038/s41598-017-16654-1 (PMC5705678; doi:10.1038/s41598-017-16654-1)
Supplement: Supplementary file 1 — Supplementary Information [file 41598_2017_16654_MOESM1_ESM.pdf]

Supplementary Information for

Fabrication-Method-Dependent Excited State Dynamics in $CH_3NH_3PbI_3$ Perovskite Films

In-Sik Kim[1], Cheol Jo[1], Rira Kang[2], Dong-Yu Kim[3], Seong-Jin Son[1], In-Wook Hwang[4], and Do-Kyeong Ko[1,4,*]

[1]Department of Physics and Photon Science, Gwangju Institute of Science and Technology, Gwangju 61005, Republic of Korea.
[2]Radiation Research Division for Industry & Environment, Korea Atomic Energy Research Institute, Jeongeup, Jeollabuk-do 56212, Republic of Korea.
[3]School of Materials Science and Engineering, Gwangju Institute of Science and Technology, Gwangju 61005, Republic of Korea.
[4]Advanced Photonics Research Institute, Gwangju Institute of Science and Technology, Gwangju 61005, Republic of Korea.
[*]Correspondence and requests for materials should be addressed to D.-K.K. (e-mail: dkko@gist.ac.kr)

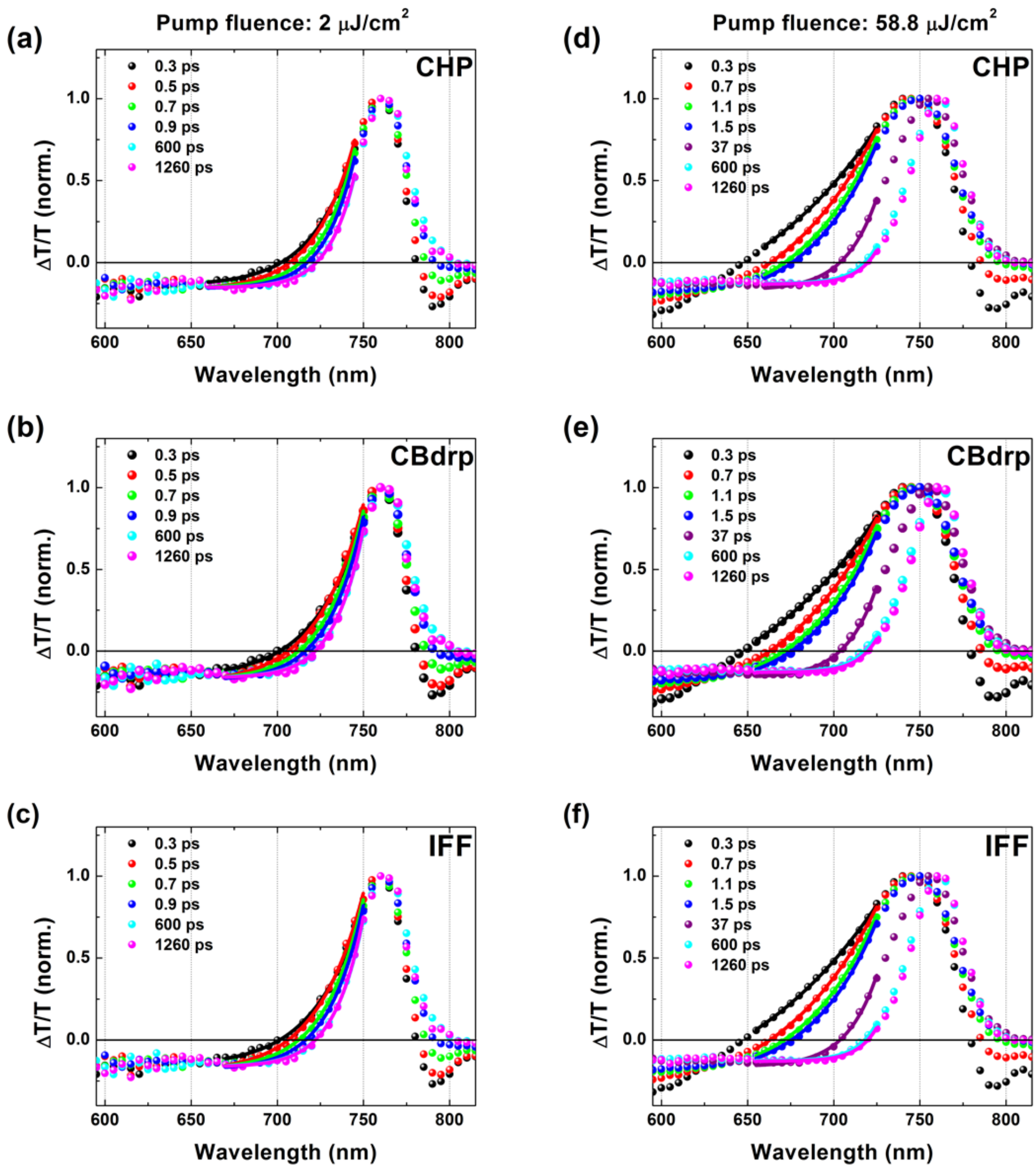

Figure S1. Normalised transient absorption (TA) spectra (symbols) of $CH_3NH_3PbI_3$ films fabricated by three fabrication (CHP, CBdrp, and IFF) methods, recorded at several pump-probe delays after 400-nm excitation at the fluence of (a)-(c) 2 μJ cm^{-2} and (d)-(f) 58 μJ cm^{-2}. The photo-bleaching region from ~660 nm to ~730 nm of each spectrum was fitted by a reduced Maxwell–Boltzmann distribution function (lines) of $\exp(-E/k_BT_c)$, where k_B is Boltzmann's constant and energy E is significantly higher than Fermi level. The carrier temperature (T_c) as a result of the fit is depicted in Fig. S2.

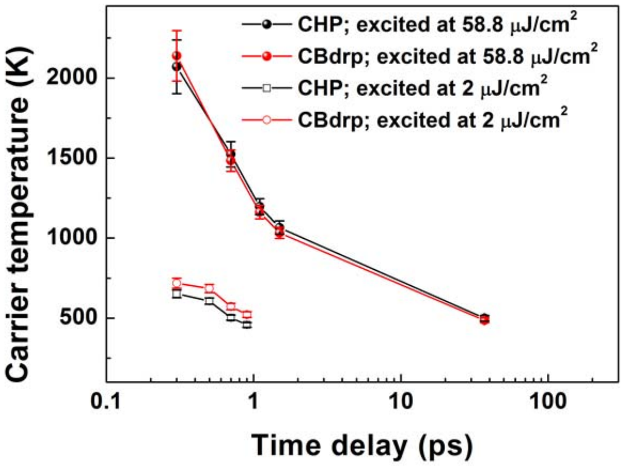

Figure S2. Carrier temperature dynamics in CHP (black) and CBdrp (red) films at low and high excitation fluences. Error bars indicate the deviation between the photo-bleaching spectra and the Maxwell-Boltzmann function. The results for the IFF film were almost identical to those for the CBdrp film and were thus excluded from this figure.

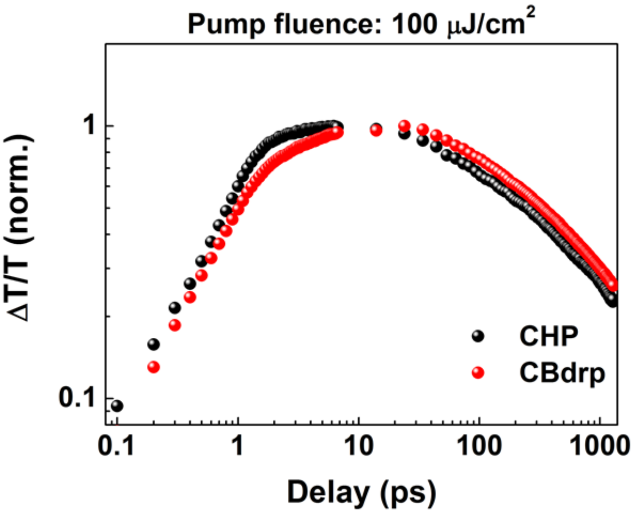

Figure S3. Normalised TA decay kinetics in CHP (black) and CBdrp (red) films, probed at 760 nm after 400 nm excitation with the fluence of 100 μJ cm^{-2}.

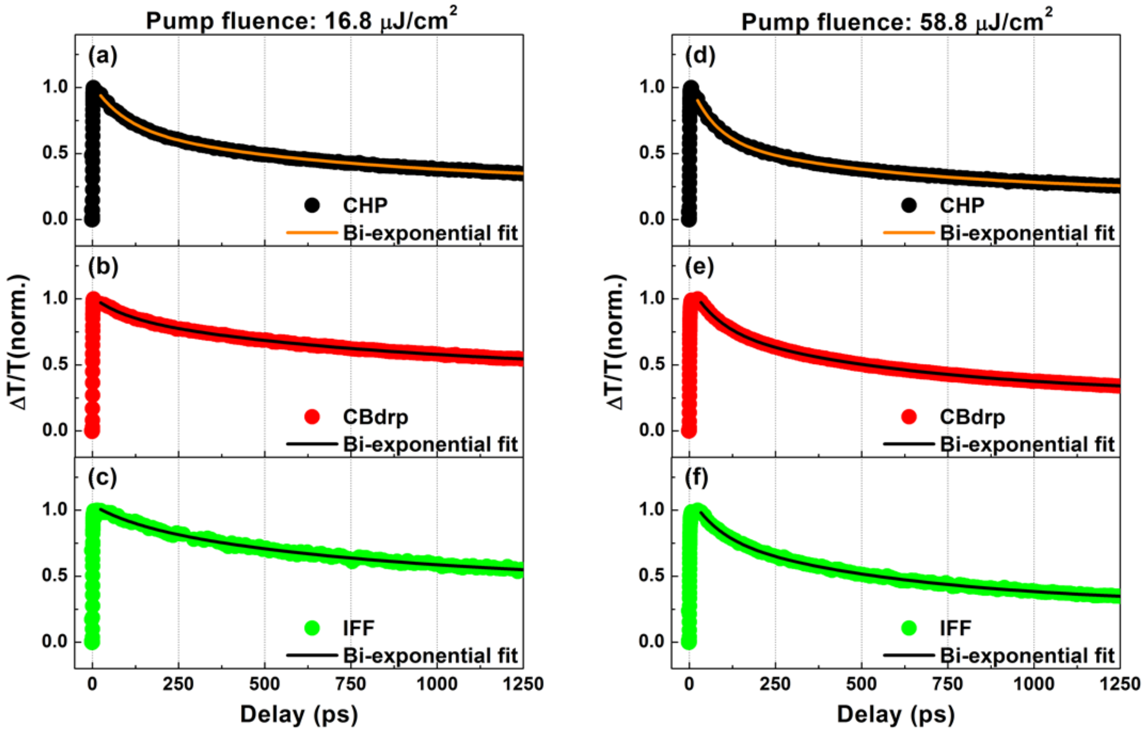

Figure S4. Normalised 760-nm kinetics of each perovskite with 400-nm excitation at high fluences of (a)-(c) 16.8 μJ cm^{-2} and (d)-(f) 58.8 μJ cm^{-2}. The solid lines in each panel are the bi-exponential fits for the TA data.

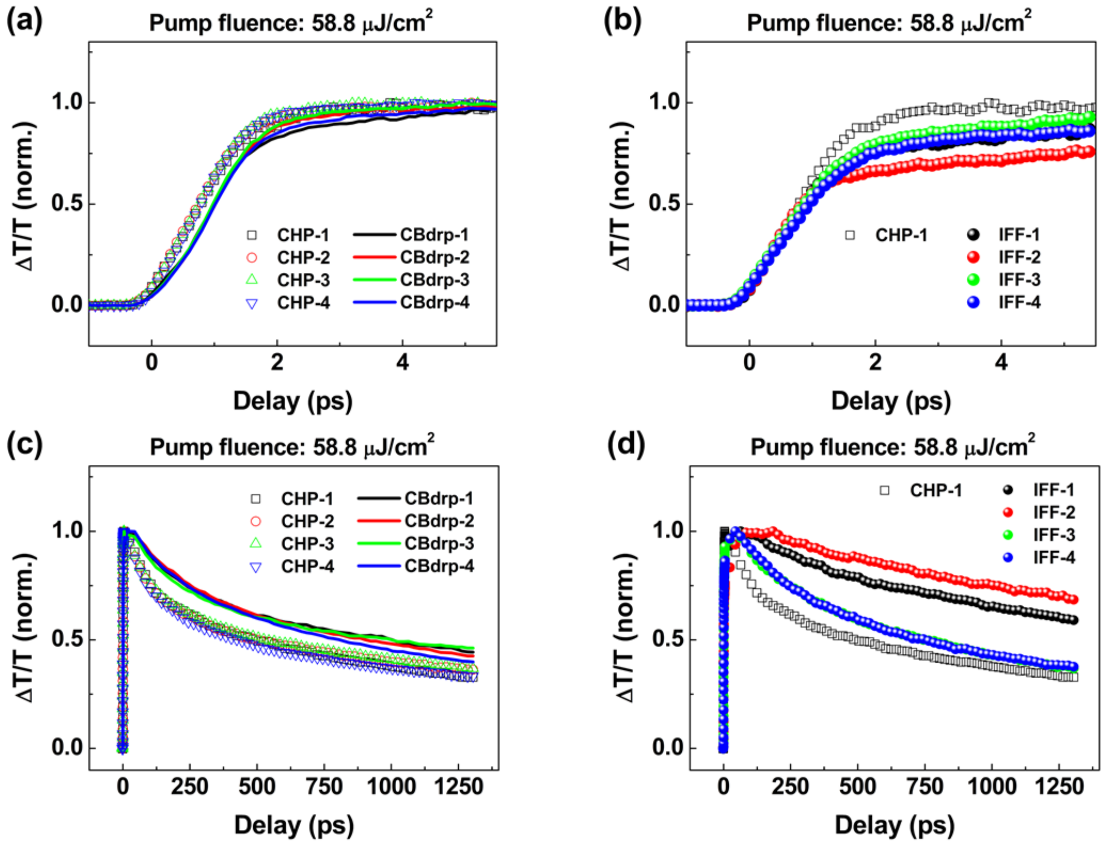

Figure S5. The comparison of normalized 760 nm-kinetics of additionally prepared films in (a),(b) short and (c),(d) long delay ranges. The excitation wavelength and fluence were 400 nm and 58.8 μJ cm^{-2}, respectively.

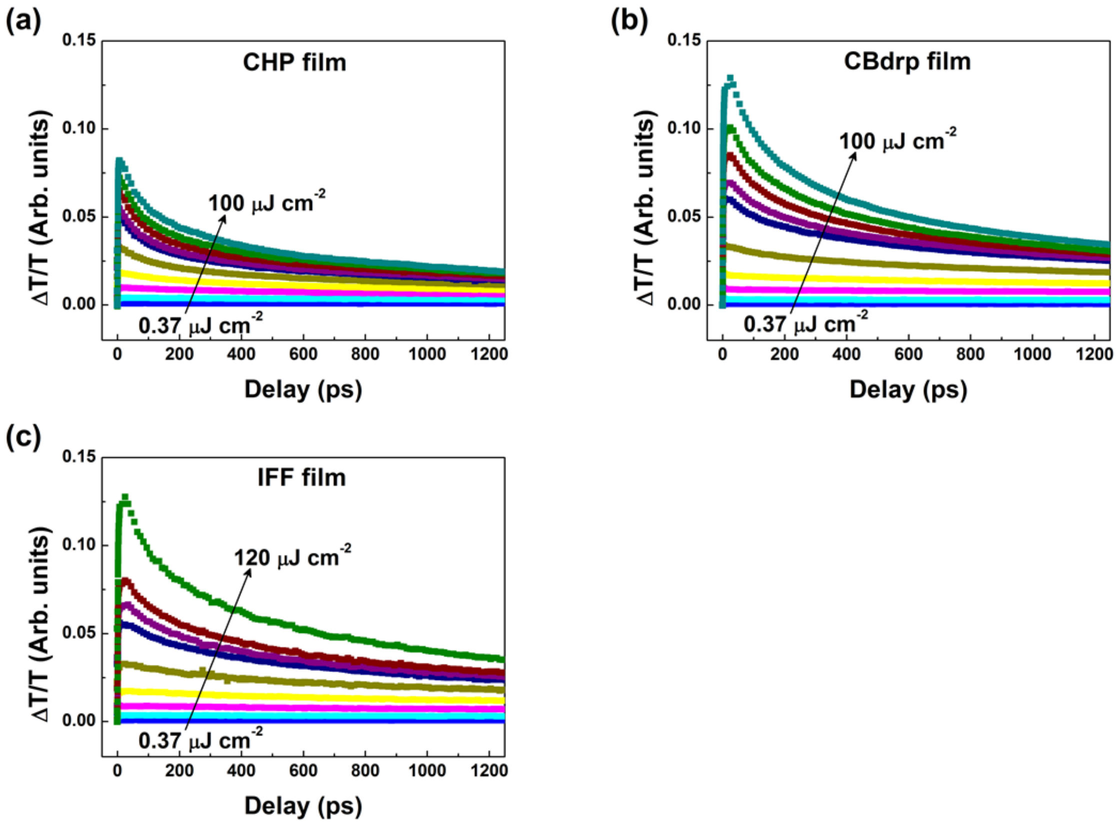

Figure S6. Kinetic profiles of (a) CHP, (b) CBdrp, and (c) IFF films, probed at 760 nm excited with various fluences of 400-nm excitation. Each profile was obtained by averaging of multiple decay data for just one film at each given excitation condition.
